# How well do radiographic, clinical and self-reported diagnoses of knee osteoarthritis agree? Findings from the Hertfordshire cohort study

**DOI:** 10.1186/s40064-015-0949-z

**Published:** 2015-04-15

**Authors:** Camille Parsons, Michael Clynes, Holly Syddall, Darshan Jagannath, Anna Litwic, Suzan van der Pas, Cyrus Cooper, Elaine M Dennison, Mark H Edwards, the EPOSA research group

**Affiliations:** MRC Lifecourse Epidemiology Unit, University of Southampton, Southampton General Hospital, Southampton, SO16 6YD UK; Department of Epidemiology and Biostatistics, EMGO Institute for Health and Care Research, VU University Medical Centre, Amsterdam, the Netherlands; NIHR Biomedical Research Centre, University of Southampton and University Hospital Southampton NHS Foundation Trust, Southampton, SO16 6YD UK; NIHR Musculoskeletal Biomedical Research Unit, University of Oxford, Oxford, OX3 7LE UK

**Keywords:** Osteoarthritis, Epidemiology, Agreement, Radiographic, Definition

## Abstract

**Objective:**

Epidemiological studies of knee osteoarthritis (OA) have often used a radiographic definition. However, the clinical syndrome of OA is influenced by a broad range of factors in addition to the structural changes required for radiographic OA. Hence more recently several studies have adopted a clinical or self-reported approach to OA diagnosis rather than a radiographic approach. The aim of this study was to investigate agreement between radiographic OA and the clinical and self-reported diagnoses of OA.

**Design:**

Data were available for 199 men and 196 women in the Hertfordshire Cohort Study (HCS), UK. Participants completed a questionnaire detailing self-reported OA. Clinical OA was defined based on American College of Rheumatology (ACR) criteria. Knee radiographs were taken and graded for overall Kellgren and Lawrence (K&L) score.

**Results:**

The mean (standard deviation (SD)) age of study participants was 75.2 (2.6) years and almost identical proportions of men and women. The prevalence of knee OA differed depending on the method employed for diagnosis; 21% of the study participants self-reported knee OA, 18% of the participants had clinical knee OA and 42% of the participants had radiographic OA. Of those 72 study participants with a self-reported diagnosis of knee OA 52 (72%) had a radiographic diagnosis of knee OA, while 66% (39 out of 59) of study participants with clinical knee OA had a diagnosis of radiographic knee OA. However 58% of those participants diagnosed with radiographic OA did not have either self-reported knee OA or a diagnosis of clinical OA. Therefore in comparison with the radiographic definition of OA, both the clinical and self-report definitions had high specificity (91.5% & 91.5% respectively) and low sensitivity (24.5% and 32.7% respectively).

**Conclusion:**

There is modest agreement between the radiographic, clinical and self-report methods of diagnosis of knee OA.

## Introduction

Osteoarthritis (OA) is the most prevalent joint disease in older adults (Lawrence et al. 2008; Vos et al. 2012) and it has been estimated that 40% of the population aged over 65 years is affected by knee or hip symptomatic OA (Dawson et al. 2004). OA is a degenerative disease that affects the structures within the affected joint. During the natural disease progression of OA the breakdown of cartilage occurs which then leads to subchondral bone and formation of osteophytes, deterioration of tendons and ligaments surrounding the affected joint and varying levels of synovitis (Litwic et al. 2013).

Epidemiological studies of knee OA have often been based on a radiographic definition of knee OA (Cooper et al. 2000) to capture the structural changes in the joint of interest, and most studies employ the radiographic technique first proposed by Kellgren and Lawrence (1957a), which characterises knee OA into four grades (0, normal to 4, severe). Conventionally, an individual is classified as suffering from knee OA if their knee radiograph is scored as Kellgren and Lawrence grade 2 or above (Dennison and Cooper 2003).

However a disadvantage of defining OA based on radiographic data alone is that the clinical syndrome of OA is influenced by a broad range of factors in addition to structural changes. For example, researchers have shown that joint pain in OA is heightened by co-morbid illness, muscle-strength, mood, cognition and disability (Issa and Sharma 2006). Hence a radiological approach may not accurately reflect the clinical burden of the condition.

An alternative approach to assessing the prevalence of OA is therefore to make a diagnosis based on clinical criteria. In the early 1990’s the American Rheumatism Association (ACR) developed a definition of OA that is based on the clinical characteristics of individuals (Altman 1991). The ACR’s clinical approach to defining OA takes into account medical history, laboratory test results and physical examination to identify knee OA rather than using radiographic images to obtain a diagnosis.

Finally, in addition to radiographic or clinical definitions of knee OA, some epidemiological studies have implemented a self-reported, subjective, definition of knee OA (Thomas et al. 2014; Van der Pas et al. 2013). In these instances, study participants have been asked to self-report whether they believe they have OA in the joint of interest, by being asked such questions as ‘Do you have OA?’ or ‘Have you had any pain in your (joint region) over the last year?’

Little is known about the agreement between the radiographic, clinical and self-reported definitions of OA. Therefore the aim of this study was to investigate the agreement between radiographic OA and the clinical and self-reported diagnoses of OA among community dwelling older men and women who participated in the Hertfordshire Cohort Study (HCS), UK.

## Methods

### Study design

The study sample comprised men and women who participated in the UK component of the European Project on Osteoarthritis (EPOSA) and, who originally participated in the HCS; the EPOSA and HCS studies have been described in detail previously (Van der Pas et al. 2013; Syddall et al. 2005). In brief, HCS is a large, prospective, population-based study of the lifecourse origins of adult disease among men and women born in Hertfordshire between 1931 and 1939 and still living in the county between 1998 and 2004. A total of 592 HCS participants were eligible to participate in EPOSA, of whom 444 (75%) provided written informed consent to participate in the study. EPOSA participants were visited at home by a trained research nurse, who administered a questionnaire which incorporated the Western Ontario and McMaster Universities Osteoarthritis Index (WOMAC) – a 24-item questionnaire with 3 subscales measuring pain, stiffness and physical function (Bellamy et al. 1988).

Study participants were asked “Do you have osteoarthritis?” and if the response was “yes” the joint affected by OA was ascertained, with the focus of this study being knee OA.

During the EPOSA home visit a clinical examination of OA was also conducted. The ACR classification was used to identify clinical OA among the EPOSA participants (Altman 1991). In brief, a clinical diagnosis of knee OA was made if a study participant reported pain in the knee (as evaluated by the WOMAC pain subscale), plus any 3 of: age over 50 years; morning stiffness in the knee lasting <30 minutes (evaluated by the WOMAC stiffness subscale); crepitus on active motion in at least one side; bony tenderness; bony enlargement or no palpable warmth of synovium.

Anterio-patellofemoral (AP) and lateral knee x-rays were taken of both knees at a local hospital after the home visit and the knee joints were graded based on the Kellgren and Lawrence (K&L) score (Kellgren and Lawrence 1957a). The K&L grading system is briefly described as follows: grade 1: unlikely narrowing of the joint space and possible osteophytes on the radiograph; grade 2: small osteophytes and possible narrowing of the joint space; grade 3: Multiple, moderately sized osteophytes, definite joint space narrowing, some sclerotic areas and possible deformation of bone ends; grade 4: Multiple large osteophytes, severe joint space narrowing, marked sclerosis and definite bony end deformity (Kellgren and Lawrence 1957b). The knee with the worst (highest) K&L grade was used in the study and individuals with a tiobiofemoral joint score of 2 or more were classified as having radiographic OA.

### Statistical analysis

Characteristics of study participants were described using means and standard deviations (SD) for continuous variables and frequencies and percentages for binary and categorical variables. Gender differences were analysed using the *t*-test for continuous variables and either chi-squared or Fisher’s exact tests for categorical variables.

To assess the strength of agreement between the radiographic and clinical definitions of OA, and between radiographic and self-reported definitions of OA, sensitivity, specificity and relative risk statistics were calculated. This approach regards a radiographic diagnosis of OA as the ‘gold standard’. Sensitivity is the proportion of those with radiographic diagnosis of OA who also have a positive OA diagnosis according to the alternative definition of interest (i.e. clinical or self-reported). Specificity is the proportion of those with a negative radiographic diagnosis of OA who also have a negative OA diagnosis according to the alternative definition of interest. Relative risk (RR) is the ratio of the risk of having an OA diagnosis according to the alternative definitions of interest in individuals with and without radiographic OA.

## Results

The characteristics of the 395 study participants who had diagnosis details captured for all three OA definitions are described in Table 1. The mean age of the study participants at EPOSA baseline questionnaire was 75.2 years (SD 2.6 years). Men were on average taller and heavier than women but had similar body mass index (BMI) and level of education. Men were more likely to report ever having smoked and currently consuming alcohol.

**Table 1:** **Participant characteristics**

	**Males**		**Females**		**p-value** ^**2**^
	**(** ***n = 199)***		***(n = 196)***		
	**Mean**	**SD**	**Mean**	**SD**	
Age (years)	75.0	2.5	75.2	2.6	0.39
Height (cm)	173.0	6.5	158.9	6.0	<0.001
Weight (Kg)^1^	81.5	1.1	73.7	1.2	<0.001
BMI (kg/m^2^)^1^	27.1	1.1	27.1	1.2	0.26
	**N**	**%**	**N**	**%**	
Smoker status					
Never smoked	80	40.2	125	63.8	
Current/Ex-smoker	119	59.8	71	36.2	<0.001
Consumes alcohol (yes)	187	94.0	134	68.4	<0.001
Education					
Primary or less	40	20.1	38	19.4	
Secondary	101	50.8	84	42.9	
College and/or University	58	29.1	74	37.8	0.17
Kellgren and Lawrence scale score					
0	56	28.1	43	21.9	
1	67	33.7	70	35.7	
2	62	31.2	70	35.7	
3	12	6.0	12	6.1	
4	2	1.0	1	0.5	0.64
Radiographic OA (yes)	76	38.2	83	42.3	0.40
Clinical Knee OA (yes)	23	11.6	36	18.4	0.06
Self-reported knee OA (yes)	31	15.6	41	20.9	0.17

^1^Geometric mean and SD.

^2^p-value for differences between genders.

Prevalence of knee OA differed greatly depending on the definition used to diagnose knee OA; 21% of women and 16% of men self-reported knee OA (p = 0.17 for gender difference); 12% of men and 18% of women had a diagnosis of clinical knee OA (p = 0.06 for gender difference), and 38% of men and 42% of women had a radiographic diagnosis of knee OA (p = 0.40 for gender difference). Women were more likely to be diagnosed with knee OA than men according to the clinical or radiographic definitions but the proportions of men and women self-reporting knee OA were similar.

**Figure 1:**
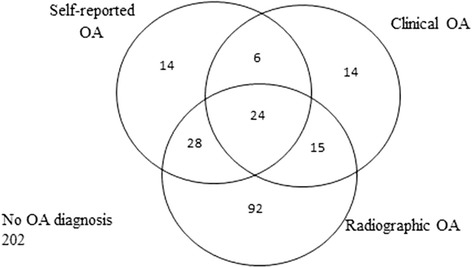
Venn diagram showing overlap between definitions of self-reported OA, clinical OA and radiographic OA in the knee.

Figure 1 demonstrates the overlap between the different definitions of OA. Of the 72 participants with self-reported knee OA, 52 (72.2%) also had radiographic knee OA. Of the 59 participants with clinical knee OA, 39 (66.1%) also had radiographic knee OA.

Table 2 presents sensitivity, specificity, positive predictive values, negative predictive values and relative risk statistics for agreement between clinical knee OA and radiographic OA, and self-reported OA and radiographic knee OA. Both the clinical and self-report definitions can be considered very good at correctly identifying those without knee OA, since both have high specificity values of 91.5% and 91.5% respectively. Conversely both the clinical and self-report definitions have low sensitivity values, 24.5% and 32.7% respectively. Those with a diagnosis of clinical knee OA were 1.85 times as likely as those without clinical knee OA to have a diagnosis of radiographic knee OA, while those with a diagnosis of self-reported knee OA are over twice as likely to have radiographic knee OA than those without a self-report diagnosis of knee OA.

**Table 2:** **Agreement between radiographic and clinical knee OA, and radiographic and self-reported knee OA**

	**Clinical knee OA**		**Self-reported OA**	
	**OA positive**	**OA negative**	**OA positive**	**OA negative**
**Radiographic OA**				
OA Positive	39	120	52	107
OA Negative	20	216	20	216
Sensitivity	24.5%		32.7%	
Specificity	91.5%		91.5%	
Positive Predictive Value	66.1%		72.2%	
Negative Predictive Value	64.3%		66.9%	
Relative Risk (95% CI)	1.85 (1.47 – 2.33)		2.18 (1.77 – 2.69)	

## Discussion

The aim of this study was to investigate levels of agreement between a radiographic diagnostic approach for knee OA and both clinical and self-reported diagnoses of knee OA. We found modest associations between radiographic knee OA and clinical knee OA (sensitivity and specificity of 24.5% and 91.5% respectively, RR of 1.85 (95% CI 1.47 – 2.33)) and between radiographic knee OA and self-reported knee OA (sensitivity and specificity of 32.7% and 91.5% respectively, RR of 2.18 (1.77 – 2.69)).

In this study an overall definition of clinical OA was used to assess agreement with radiographic OA whereas previous studies have assessed the relationship between component parts of the clinical OA algorithm and radiographic OA (Claessens et al. 1990; Felson et al. 1997). Claeseens et al. at looked at 18 clinical components of clinical OA (including pain and swelling) in a population of 2865 individuals from the Netherlands. They found a significant association between 14 of the 18 clinical components and radiographic OA (all but Heberden’s nodes, palpable knee effusion, pain in both hands, and latex fixation test) in the knee (Claessens et al. 1990). Ultimately Claeseens at al concluded that the strength of association with the component clinical findings was insufficient for any single clinical component finding to predict radiographic OA. Felson at al assessed the association between a clinical definition of OA and radiographic OA and found reasonable associations to exist (sensitivity of 53.8% and specificity of 77.9%) when considering clinical OA as the gold standard (Felson et al. 1997). Hence our findings based on a summary marker of clinical OA and using radiographic OA instead of clinical OA as the standard definition are broadly consistent with those of Claeseens et al. and Felson et al.

A substantial proportion of men and women in our EPOSA cohort were diagnosed with radiographic OA but did not have self-reported OA or a diagnosis of clinical OA (57.7%). This is consistent with previous studies that have shown radiographic knee OA correlates poorly with the physical symptoms of OA (Hannan et al. 2000). Thus, it can be argued that making a comparison between studies that have used the radiographic definition of OA alone with studies employing clinical or self-reported definitions of OA is problematic.

Moreover, in the clinical setting where the focus of intervention is the improvement of symptoms, the use of a self-reported or clinical definition of knee OA may be of more interest than a radiographic definition. Although a radiographic definition of knee OA may highlight structural changes within the knee, for example at an early stage of disease, if symptoms are yet to be experienced by the patient it is unlikely that knee OA identified only through radiographs has yet affected a patient’s physical functioning and quality of life. Other factors such as inflammatory mediators and markers of cartilage degradation can be considered as part of diagnostic tools for knee OA in research, however these are seldom used in clinical practice, and therefore have not be considered in the current study.

This study has some limitations. First, a ‘healthy’ responder bias is unsurprisingly evident within the Hertfordshire Cohort Study of which EPOSA is a subset cohort (Breslow and Day 1987). However, the cohort has been shown to be broadly comparable with participants in the nationally representative Health Survey for England (Syddall et al. 2005) and a healthy responder bias is unlikely to have affected the inter-relationship between different OA definitions. In this study a slightly higher prevalence of self-reported knee OA maybe present than in other studies. During EPOSA a study questionnaire asking about self-reported OA was administered before WOMAC or clinical examinations. However some 15 years ago the same study subjects had knee x-rays as part of the initial Hertfordshire Cohort Study, leading to the possibility of some recall bias. Our study also has many strengths, including extensive phenotyping of study participants according to strict study protocols, and by a highly trained research team.

In conclusion, there is only modest agreement between the radiographic, clinical and self-report methods of diagnosis of knee OA. Further research is required to identify the optimal method of diagnosis of knee OA for use in research settings.
